# Circulating tumor cells undergoing EMT are poorly correlated with clinical stages or predictive of recurrence in hepatocellular carcinoma

**DOI:** 10.1038/s41598-019-43572-1

**Published:** 2019-05-08

**Authors:** Yunyang Chen, Shaoming Li, Wei Li, Rongbing Yang, Xianguang Zhang, Yong Ye, Jiexiong Yu, Lin Ye, Wangrong Tang

**Affiliations:** Department of Hepatobiliary Surgery, Jiangmen Central Hospital, Affiliated Jiangmen Hospital of Sun Yat-Sen University, Jiangmen 529000, Guangdong, China

**Keywords:** Tumour biomarkers, Hepatocellular carcinoma

## Abstract

Experimental and clinical studies have highlighted that circulating tumor cell (CTC) with phenotypic hallmarks of epithelial-mesenchymal transition (EMT) plays a critical role in the metastatic and recurrence of solid malignancy. Here we retrospectively evaluated the presence of CTC and its EMT phenotypes in hepatocellular carcinoma (HCC) patients and investigated their clinical relevance. We optimized the Canpatrol^TM^ CTC analysis system to enumerate CTC and classify EMT phenotypes in 113 HCC patients before curative treatment and 143 HCC patients after curative treatment. The relationships between CTC and clinical characteristics were statistically analyzed. None of total CTC or its EMT phenotypes in HCC patients was correlated with clinical characteristics, such as age, sex, HBsAg, Child-Pugh score, liver cirrhosis, AFP, number of tumors, tumor size, vascular invasion and BCLC stage. Neither the level of total CTC nor its EMT phenotypes in HCC patients before or after curative treatment were predictive of recurrence. Additionally, dynamic changes of CTC and its EMT phenotypes were not relevant to HCC recurrence after curative treatment in our study. Wefound CTC count and EMT classification were not correlated with clinical stages or predictive of HCC recurrence, but further large, multicenter studies are needed to confirm these results.

## Introduction

Hepatocellular carcinoma (HCC) is one of the most common cancers worldwide, with over half a million new cases diagnosed annually and the mortality rate rapidly increased. Curative therapeutic options for HCC patients are radical surgical resection, local ablation or liver transplantation, but limited to a small proportion of patients with early-stage HCC. The outcome is not always satisfactory because of a high incidence of HCC recurrence after curative therapy, which contributes to high mortality rates^1^. However, current imaging tests, common serum markers and pathological features are lack of accuracy and sensitivity in prediction and detection of early recurrence^1,2^. Therefore, novel and reliable approaches are necessary to be found to monitor recurrence for HCC patients.

Circulating tumor cell (CTC) has been defined as cancer cell released into the blood circulation from solid tumor origin, which represent viable metastatic precursor cell capable of initiating a clonal metastatic lesion. In current hypothesis, the presence of CTC represents a strong independent prognostic factor for reduced disease-free and overall survival in multiple epithelial tumor types^3–5^. During the dissemination of cancer cells, epithelial cells frequently exhibit a downregulation of epithelial markers and a loss of intercellular junctions. The loss of epithelial features is often accompanied by increased expression of mesenchymal genes. This process, described as epithelial-mesenchymal transition (EMT), endows cancer cells with migratory and invasive properties and promotes cancer recurrence^6,7^. Recent reports addressing the correlation between EMT-markers expression in CTC and cancer progression have revealed that EMT of CTC is a relevant process for invasion and metastasis in breast cancer, non-small cell lung cancer, prostate cancer, gastric cancer, colorectal cancer, and so on^8–12^. As well in HCC, several studies have demonstrated that the CTC count and EMT-markers expression are of great relevance with clinicopathological features^13–16^. However, the results are with great heterogeneity due to different detection methods of CTC and EMT-markers, and the small sample sizes compromise the statistical power. Especially, prognostic value of CTC count and EMT-markers for recurrence in HCC patients after curative treatment needs further investigation.

In the present study, we optimized the Canpatrol^TM^ CTC analysis system to enumerate CTC and classify EMT phenotypes, and retrospectively investigated the clinical relevance, dynamic changes, and prognostic significance of these cells in HCC patients before and after curative treatment.

## Results

### Clinical characteristics and correlation with CTC count

Peripheral blood samples were collected from 113 HCC patients before any curative treatment, including tumor resection or ablation. The clinical characteristics and CTC prevalence of the 113 pre-treated HCC patients were listed in Table 1. For all patients, total CTC ≥3 per 5 ml blood were detected in 89/113 patients (78.8%) and the average number was 11.2 (range, 0 to 75). The positive rate (≥1 per 5 ml blood) of the epithelial, biophenotypic, and mesenchymal CTC was 46.0% (52/113), 86.7% (98/113) and 50.4% (57/113), respectively. The average number of epithelial, biophenotypic and mesenchymal CTC was 1.6 (range, 0 to 20), 7.6 (range, 0 to 66), and 1.9 (range, 0 to 18), respectively. The presence of total CTC did not significantly correlate with any of the clinical characteristics, including age (*P* = 0.088), sex (*P* = 0.778), HBsAg (*P* = 0.863), Child-Pugh score (*P* = 0.138), liver cirrhosis (*P* = 0.754), level of AFP (*P* = 0.757), number of tumors (*P* = 0.225), tumor size (*P* = 0.192), vascular invasion (*P* = 0.614) or BCLC stage (*P* = 0.189). Neither significant correlation was observed between epithelial, biophenotypic, or mesenchymal CTC count and the above clinical characteristics (Table 1).

**Table 1:** Clinical characteristics and correlation with CTC count in 113 pre-treated HCC patients.

Clinical Characteristics (N = 113)	Total CTC			Epithelial CTC			Biophenotypic CTC			Mesenchymal CTC		
	≥3	<3	*P*	≥1	<1	*P*	≥1	<1	*P*	≥1	<1	*P*
All	89	24		52	61		98	15		57	56	
Age, years			0.088			0.238			0.113			0.280
>50	58	20		33	45		65	13		42	36	
≤50	31	4		19	16		33	2		15	20	
Sex			0.778			0.297			0.801			0.982
Male	84	23		48	59		93	14		54	53	
Female	5	1		4	2		5	1		3	3	
HBsAg			0.863			0.992			0.812			0.794
Negative	10	3		6	7		11	2		7	6	
Positive	79	21		46	54		87	13		50	50	
Child-Pugh score			0.138			0.085			0.994			0.754
A	75	23		42	56		85	13		50	48	
B	14	1		10	5		13	2		7	8	
Liver cirrhosis			0.754			0.383			0.698			0.780
No	44	11		23	32		47	8		27	28	
Yes	45	13		29	29		51	7		30	28	
AFP			0.757			0.209			0.510			0.064
<20 ng/mL	34	10		17	27		37	7		27	17	
≥20 ng/mL	55	14		35	34		61	8		30	39	
No. of tumors			0.225			0.449			0.447			0.500
Single	47	16		27	36		56	7		30	33	
Multiple	42	8		25	25		42	8		27	23	
Tumor size			0.192			0.973			0.725			0.109
<5 cm	35	13		22	26		41	7		20	28	
≥5 cm	54	11		30	35		57	8		37	28	
Vascular invasion			0.614			0.410			0.856			0.957
No	66	19		41	44		74	11		43	42	
Yes	23	5		11	17		24	4		14	14	
BCLC stage			0.189			0.440			0.918			0.290
0 + A	28	11		16	23		34	5		17	22	
B + C	61	13		36	38		64	10		40	34	

### Prevalence of CTC count before curative treatment and their prognostic value for recurrence

**Figure 1:**
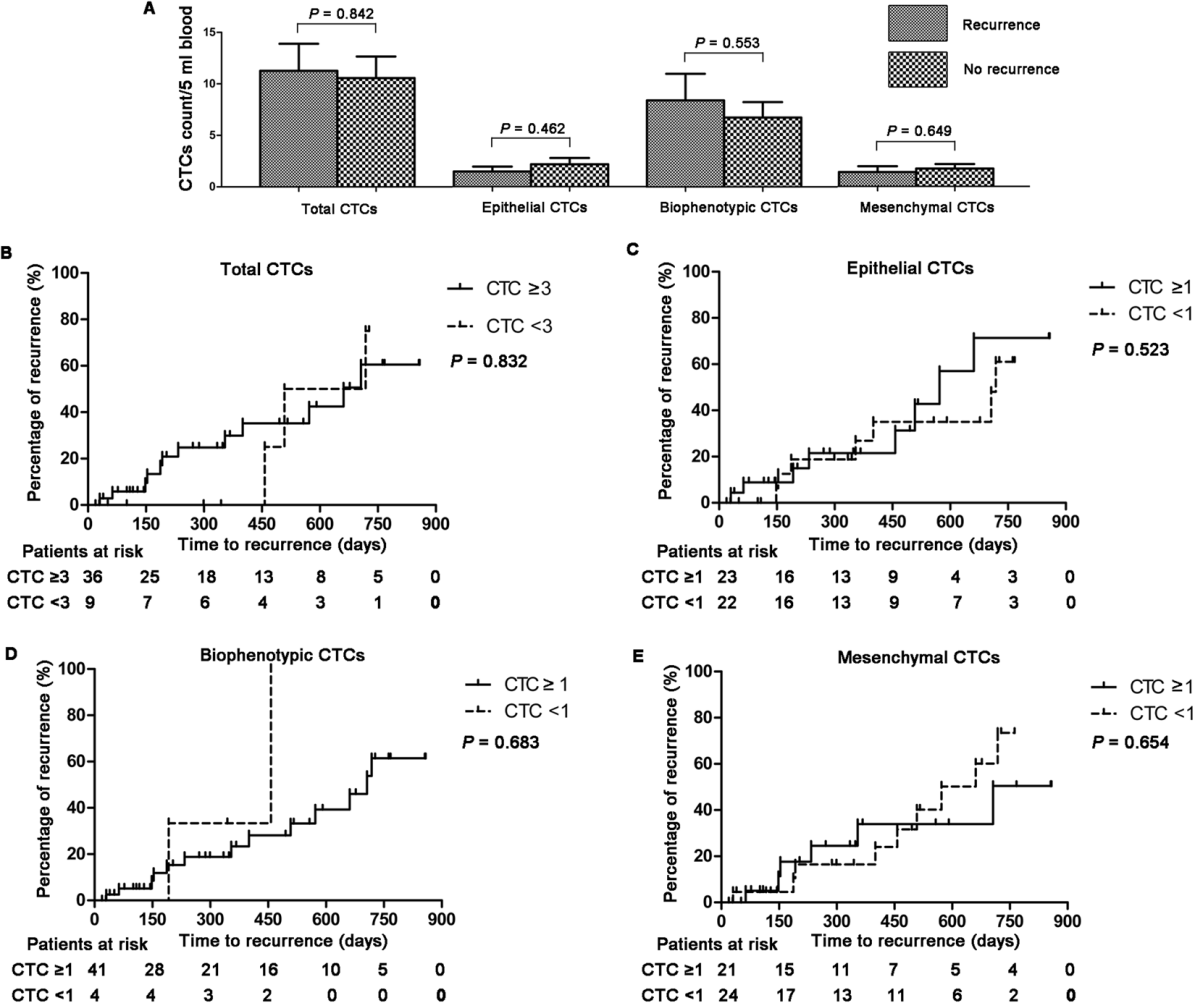
Prevalence of CTC count before curative treatment and their prognostic value for recurrence in 45 HCC patients. (**A**) Mean numbers of total CTC, epithelial CTC, biophenotypic CTC, and mesenchymal CTC before curative treatment in recurrent patients versus non-recurrent patients. (**B**–**E**) Kaplan-Meier analysis for time to recurrence in HCC patients with total CTC ≥3 versus <3 (**B**), epithelial CTC ≥1 versus <1 (**C**), biophenotypic CTC ≥1 versus <1 (**D**), mesenchymal CTC ≥1 versus <1 (**E**).

Among the 113 HCC patients, 45 had been treated with radical surgical resection, radio frequency or microwave ablation, which were considered as curative treatment, and blood samples for CTC test were collected before that. By the time of analysis, 15 of 45 patients had been diagnosed with recurrent HCC with an average interval to recurrence of 358.8 ± 60.7 days (median, 354 days; range, 30 to 718 days). Total CTC count in recurrent cases was not significantly greater than those in non-recurrent cases (11.2 ± 2.6 vs. 10.6 ± 2.1, *P* = 0.842, Fig. 1A). Similar results were also found in epithelial CTC (1.5 ± 0.5 vs. 2.2 ± 0.7, *P* = 0.462), biophenotypic CTC (8.4 ± 2.6 vs. 6.7 ± 1.5, *P* = 0.553), and mesenchymal CTC (1.4 ± 0.6 vs. 1.7 ± 0.4, *P* = 0.649) comparing recurrent cases with non-recurrent cases (Fig. 1A).

Total CTC ≥3 per 5 ml blood were detected in 12/15 (80.0%) HCC recurrent patients, while in 24/30 (80.0%) non-recurrent patients (*P* = 1.000, Table 2). The positive rate (≥1 per 5 ml blood) of the epithelial, biophenotypic and mesenchymal CTC in HCC recurrent patients was 53.3% (8/15), 86.7% (13/15) and 40.0% (57/15), respectively. Nevertheless, the rate in non-recurrent patients was 50.0% (15/30), 93.3% (28/30) and 50.0% (15/30), respectively, which did not significantly differ from those in recurrent patients. No significant higher risk of developing postoperative recurrence was found in patients with epithelial CTC ≥1 than those with epithelial CTC <1 (*P* = 0.833), as well as in patients with biophenotypic CTC ≥1 (*P* = 0.459) and mesenchymal CTC ≥1 (*P* = 0.526) (Table 2).

**Table 2:** Prognostic value of CTC count before curative treatment for recurrence in 45 HCC patients.

	Recurrence (N = 15)	No recurrence (N = 30)	*P* value
Total CTC			1.000
≥3	12	24	
<3	3	6	
Epithelial CTC			0.833
≥1	8	15	
<1	7	15	
Biophenotypic CTC			0.459
≥1	13	28	
<1	2	2	
Mesenchymal CTC			0.526
≥1	6	15	
<1	9	15	

Among patients with total CTC ≥3, *Kaplan-Meier analysis* showed that the mean time to recurrence (TTR) was not significant shorter than those with total CTC <3 (days, 308.3 ± 66.5 vs. 561.0 ± 79.9, *P* = 0.832, Fig. 1B). Similar findings were also observed in epithelial CTC ≥1 and < 1 (days, 339.5 ± 85.0 vs. 380.9 ± 93.1, *P* = 0.523, Fig. 1C), biophenotypic CTC ≥1 and <1 (days, 364.1 ± 68.8 vs. 324.5 ± 132.6, *P* = 0.638, Fig. 1D), mesenchymal CTC ≥1 and <1 (days, 276.2 ± 94.8 vs. 413.9 ± 77.9, *P* = 0.654, Fig. 1E).

### Changes of CTC count after curative treatment and their prognostic value for recurrence

**Figure 2:**
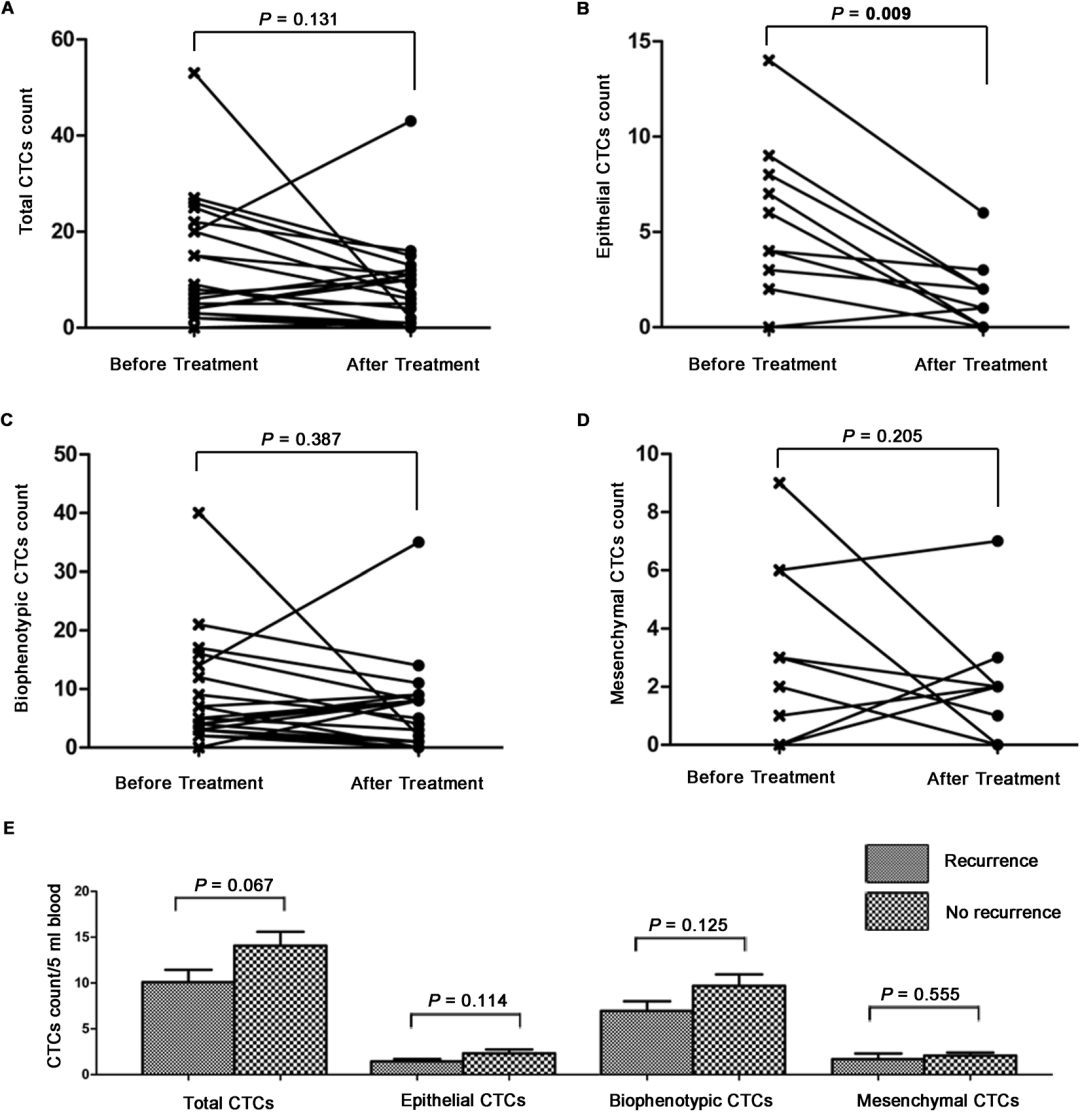
Changes of CTC count after curative treatment and their prognostic value for recurrence in 21 HCC patients. (**A**–**D**) Changes of total CTC (**A**), epithelial CTC (**B**), biophenotypic CTC (**C**) and mesenchymal CTC (**D**) count in 21 patients after curative treatment. (**E**) Mean numbers of total CTC, epithelial CTC, biophenotypic CTC, and mesenchymal CTC after curative treatment in recurrent patients versus non-recurrent patients.

Among the 113 HCC patients, CTC counts were again measured in 21 patients within 3 months after receiving tumor resection or ablation therapy. There were 14/21 (66.7%) patients showed a mean decrease of 10.4 cells per 5 ml blood in total CTC count, while 6/21 (28.6%) patients showed a mean increase of 7.7 cells per 5 ml blood and 1/21 (4.8%) patient kept in a stable level. However, in *paired t test* analysis, only epithelial CTC count had a significant mean decrease of 4.3 cells per 5 ml blood after curative treatment (*P* = 0.009, Fig. 2B). Total CTC, biophenotypic CTC and mesenchymal CTC count did not change significantly after curative treatment (*P* = 0.131, *P* = 0.387, *P* = 0.205, respectively, Fig. 2A–D).

A total of 143 HCC patients who had CTC tests after curative treatment were collected for the below analyses. By the time of analysis, 57/143 (39.9%) patients had been diagnosed with recurrent HCC. The HCC recurrent patients had similar count of total CTC (10.1 ± 1.4vs. 14.1 ± 1.5, *P* = 0.067), epithelial CTC (1.4 ± 0.3 vs. 2.3 ± 0.4, *P* = 0.114), biophenotypic CTC (6.9 ± 1.0 vs. 9.7 ± 1.3, *P* = 0.125) and mesenchymal CTC (1.7 ± 0.6 vs. 2.1 ± 0.3, *P* = 0.555) with the non-recurrent patients (Fig. 2E). Total CTC ≥3 per 5 ml blood were detected in 48/57 (84.2%) HCC recurrent patients, while in 76/86 (88.4%) non-recurrent patients (*P* = 0.473, Table 3). Analogously, the positive rate (≥1 per 5 ml blood) of the epithelial, biophenotypic and mesenchymal CTC in HCC recurrent patients and in non-recurrent patients did not differ significantly (Table 3). These results delivered the message that none of CTC and its EMT phenotypes could indicate recurrence for HCC patients after curative treatment.

**Table 3:** Prognostic value of CTC count after curative treatment for recurrence in 143 HCC patients.

	Recurrence (N = 57)	No recurrence (N = 86)	*P* value
Total CTC			0.473
≥3	48	76	
<3	9	10	
Epithelial CTC			0.066
≥1	29	57	
<1	28	29	
Biophenotypic CTC			0.928
≥1	50	75	
<1	7	11	
Mesenchymal CTC			0.383
≥1	33	56	
<1	24	30	

### Dynamic monitoring of CTC count and their prognostic value for recurrence

**Figure 3:**
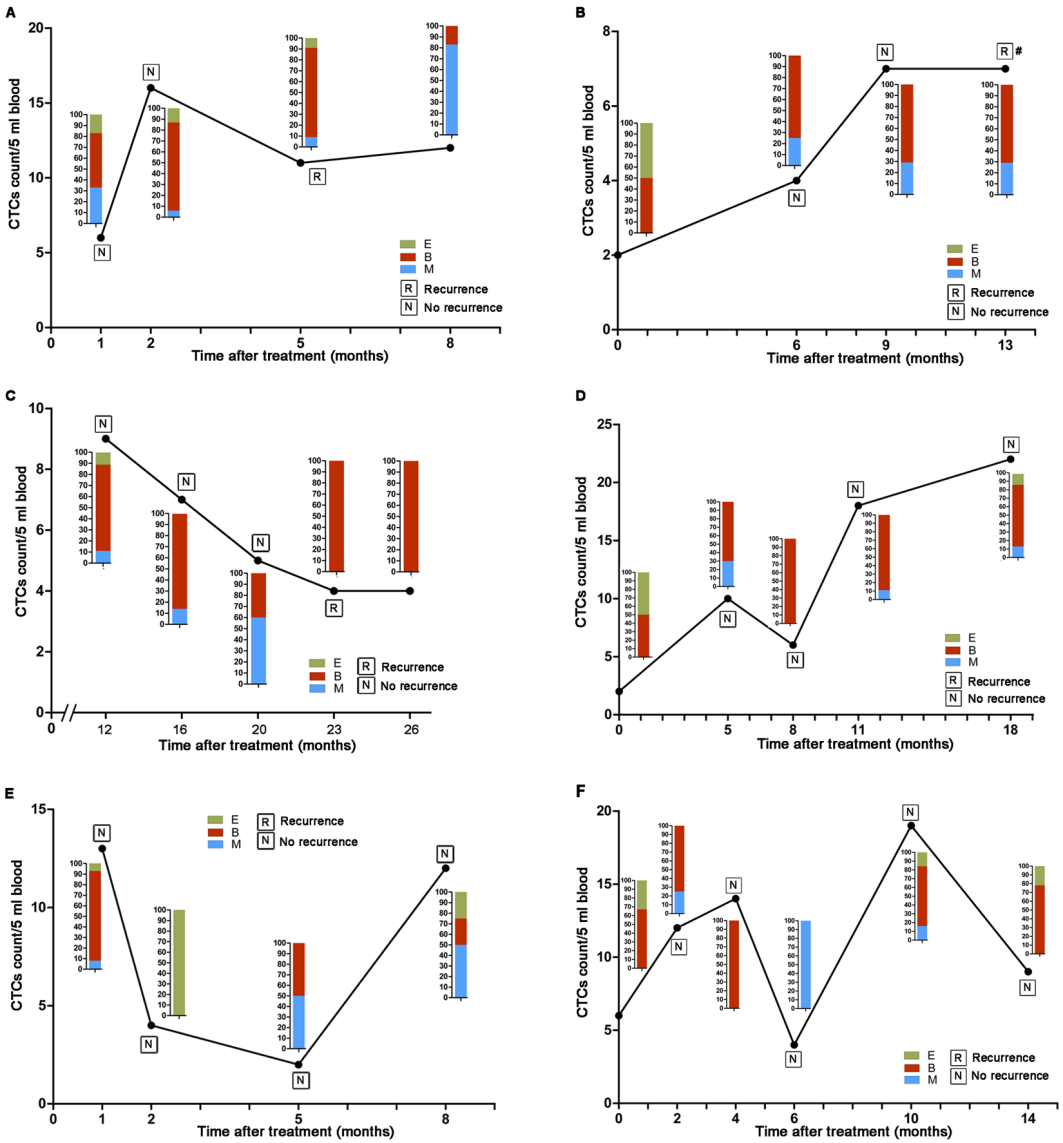
Dynamic monitoring of CTC count and their prognostic value for recurrence. (**A**) Patient A with increased CTC number, biophenotypic and mesenchymal CTC proportion showed HCC recurrence. (**B**) Patient B was diagnosed with HCC recurrence 4 months after the last CTC test. (**C**) Patient C was diagnosed with HCC recurrence with continuous decreases of CTC number. (**D**) CTC count increased continuously and biophenotypic and mesenchymal CTC accounted for most of the CTC in patient D who without recurrence. (**E**,**F**) In patient E and patient F, CTC count and proportion of biophenotypic and mesenchymal CTC increased and declined irregularly and no HCC recurrence observed.

Further investigations were performed to analyze the relation between dynamic changes of CTC and HCC recurrence. Patient A was diagnosed HCC recurrence when the CTC test presented an increase of CTC number and proportion of biophenotypic and mesenchymal CTC (Fig. 3A). Patient B shared the same experience with patient A, but differently HCC recurrence occurred 4 months after the last CTC test (Fig. 3B), which could not provide instant message about recurrence. Contrarily, patient C was diagnosed with HCC recurrence but with continuous decreases of CTC number (Fig. 3C). In patient D, CTC count raised continuously and biophenotypic and mesenchymal CTC accounted for most of the CTC, however, no recurrence occurred (Fig. 3D). In patient E and patient F, CTC count and proportion of biophenotypic and mesenchymal CTC increased and declined irregularly but without HCC recurrence observed (Fig. 3E,F). In summary, no evidence could extracted from these representative patients to prove that dynamic changes of CTC and its EMT phenotypes were relevant to HCC recurrence after curative treatment in our studies.

## Discussion

The most effective therapeutic methods for HCC at present are hepatic resection, local ablation and liver transplantation. But high mortality rates still remain mostly due to the high incidence of postoperative recurrence^1^. Experimental and clinical studies have highlighted the hypothesis that CTC play a critical role in the metastatic and recurrence of HCC^4,17–20^. In the early 1999, Vona *et al*.^21^ used the ISET (isolation by size of epithelial tumor cells) assay to detected CTC in HCC patients, which made it possible for the analysis of cell morphology, counting of tumor cells, and demonstration of tumor microemboli diffuse into peripheral blood during surgery. Later on, great enthusiasms had been devoted to develop more sensitive and specific methods for the isolation and identification of CTC in HCC patients. Fan and his colleagues^18^ used multicolor flow cytometry to detect circulating cancer stem cells (CD45^−^CD90^+^CD44^+^) in the peripheral circulation before hepatectomy, and found that patients with >0.01% circulating cancer stem cells had a lower 2-year recurrence-free survival rate and overall survival rate. A study by Sun *et al*.^4^, using the CellSearch System, detected a preoperative EpCAM^+^ CTC^7.5^ level of ≥2 is an independent prognostic indicator for HCC recurrence after curative resection, and may serve as a real-time parameter for monitoring treatment response. Recently, Canpatrol^TM^ CTC analysis system was developed to detect CTC and classify EMT phenotypes via multiple mRNA *in situ* hybridization assay, by which revealing that CTC count and EMT classification are correlated with clinical stages and metastasis of HCC^13–16^.

Nevertheless, contrary to most scholars’ positive findings, our study found that none of total CTC or its EMT phenotypes in HCC patients was correlated with clinical characteristics, such as age, sex, HBsAg, Child-Pugh score, liver cirrhosis, AFP, number of tumors, tumor size, vascular invasion and BCLC stage. Neither the level of total CTC nor its phenotypes in HCC patients before or after curative treatment were predictive of recurrence. In addition, dynamic changes of CTC and its EMT phenotypes were not relevant to HCC recurrence after curative treatment in our study. The following interpretations may help to understand the heterogeneous and contrary results.

Even though robust technologies have been developed for the isolation and identification of CTC, each method has its own advantages and disadvantages^20,22^. The nonspecific enrichment techniques utilized physicochemical CTC properties (e.g. size and density) and the specific enrichment techniques utilized markers expressed by the CTC are the two mostly used techniques. A more comprehensive discussion about advantages and disadvantages of the methods for isolation and identification of CTC can refer to Franck Chiappini^20^. To date, standardization of methodologies for CTC isolation and identification are still lacking, which make the results from dozens of studies disparate and lack statistical power, and the clinical significance of CTC in HCC patients is still controversial.

As it is known CTC are highly heterogeneous and very rare in peripheral blood; thus finding a specific marker expressed on the cell surface of every cancer cell would be of great help for CTC capture^22^. Now as yet there are no reliable markers that specifically expressed in CTC for HCC patients. Serum alpha fetoprotein (AFP) mRNA is the most important marker used in clinical routine for HCC^20^. But serum AFP mRNA can be detected during pregnancy, and in patients with acute hepatitis, chronic hepatitis, cirrhosis and other cancers. Moreover, not all HCC cells express serum AFP mRNA. By consequence, using AFP mRNA as a marker of CTC may lead to false-positive and false-negative results^20,23–25^.

As far as it goes, the cell surface markers most commonly used for CTC detection have been epithelial markers, such as EpCAM, E-cadherin and cytoketatins (CK)^26^. EpCAM positive CTC have been proposed as new prognostic biomarker reflecting the micro metastatic status and recurrence risk of HCC patients in a real-time manner^4,17^. CellSearch System, an US Food and Drug Administration (FDA)-approved device, is the most common approach for CTC detection by capturing cells expressed EpCAM^26,27^. However, the presence of EpCAM positive CTC maybe a necessary but insufficient condition for the initiation of metastasis, since CTC experience EMT process to disseminate from the primary tumor and metastasize, in which the expression of epithelial genes (e.g. EpCAM, E-cadherin and CK) will be down regulated, while expression of mesenchymal genes (e.g. vimentin and twist) will be up regulated^28,29^. In a study of breast cancer patients, significantly higher positive CTC detection rate was found using an EpCAM independent detection method compared to EpCAM-based detection technique (69.2% vs. 42.3%), indicating a loss of CTC detection by EpCAM-based detection technique^30^. EpCAM-based detection technique may therefore underestimate the actual CTC number and induce false negative results. Moreover, even though the liver is an epithelial organ,the majority of hepatocytes express EpCAM in the embryonic liver, while adult hepatocytes are EpCAM negative with only bile duct epithelium being positive in the liver tissue^31,32^. In liver neoplasia, almost all cholangiocarcinomas express EpCAM, whereas the majority of hepatocellular carcinomas are EpCAM negative, suggesting that malignant proliferation of hepatocellular carcinoma cells is not related to expression of EpCAM^32^. Taken together, the EpCAM-based detection technique, including the CellSearch system, might not be appropriate for detection of HCC CTC.

Given the limitation of EpCAM-based strategies for detection of HCC CTC, we adopted Canpatrol^TM^ CTC analysis system (Surexam Biotech, Guangzhou, China) for the isolation and identification of CTC. Briefly, blood sample was filtrated by an 8-μm-diameter-pore membrane filter, then the cells retained on the filter were stained with epithelial markers (CK 8/18/19 and EpCAM) and mesenchymal markers (vimentin and twist) by multiplex mRNA *in situ* hybridization assay^14^. For now, only a few studies with small cohort size, using the Canpatrol^TM^ CTC analysis system, suggested that CTC counts are correlated with clinical stages and metastasis of HCC^13–16^. Conversely, no significant associations were observed between CTC or its EMT phenotypes and HCC recurrence in the present study. In accordance with our study, a prospective study by Jie Zhou with 70.2% CTC positive rate also found that CTC count and subtypes were not predictive of HCC recurrence in 47 HCC patients who received liver transplantation^33^.

However, this combining size-based membrane filters and RNA-ISH technology also has its own issues. Firstly, it’s a filter-based method to isolate CTC that its disadvantages cannot deny^20,34^. Tumor cells show a wide range of sizes and go through the filter easily, especially when tumor cells experience EMT process, which might underestimate CTC count. Others types of cells in the blood might retain on the filter for the blood of some cancer patients are viscous making the results nonspecific. Cells retained on the filter can be damaged that affect later multiplex mRNA *in situ* hybridization assay. Secondly, the importance of EMT *in vivo* is fiercely debated^35^. A lack of rigorous criteria to define EMT may contribute to its ubiquitous usage in the literature. Changes in one or two genes (e.g. EpCAM, CK, twist, snail and vimentin) are often labeled by some as incomplete/partial EMT. Such genes labeled as epithelial and mesenchymal markers may be also expressed by certain epithelial, neural, inflammatory cells or normal somatic stem/progenitor cells from various organ systems^35^. In other words, as opposed to being a directed program to initiate the metastatic cascade, such changes in cancer cells may be better interpreted as the cumulative effects of the intrinsic plasticity of epithelial cells, reversion to a more primitive state and genetic instability in the context of a globally chaotic genetic background^36^. Moreover, there are numerous examples of advanced carcinomas that adopt some mesenchymal features, but otherwise retain epithelial characteristics. Fischer *et al*.^37^ established an EMT lineage tracing system to monitor this process in spontaneous breast-to-lung metastasis models. They found that lung metastases mainly consisted of non-EMT tumor cells maintaining their epithelial phenotype, and inhibiting EMT did not impact lung metastasis development. Another *in vivo* study, Zheng *et al*.^38^ also found that EMT suppression in the primary pancreatic ductal adenocarcinoma did not alter the emergence of systemic dissemination and metastasis.

The limitations of this study should be noted by the readers. This is a retrospective study with relatively small cohort size, short follow-up time, and data from a single study center. Supplementary treatments (e.g. transcatheter arterial chemoembolization, drug chemotherapy and radiotherapy) were not included in the analyses, which might influence the prognosis of the patients. Not every patient performed CTC test with standard procedure, which could probably defined as blood samples collected for CTC detection at appointed time before anticancer treatment and after curative treatment. These limitations might compromise the veracity and reliability of the results. Further large, multicenter prospective studies are needed to confirm these results as well as the effectiveness of the methods for isolation and identification of CTC.

Overall, despite the negative findings with limitations and heterogeneities found in our study, there are still numerous studies proving that CTC and its EMT phenotypes are highly associated with the tumor stage and predictive of HCC recurrence. It is without question that CTC detection and analysis have high promise for oncological drug development, monitoring the course of disease in cancer patients, and in understanding the biology of cancer progression when standard protocols are settled.

## Methods

### Patients and clinical data collection

From June 2015 to January 2018 in Jiangmen Central Hospital (Jiangmen, China), 113 HCC patients before curative treatment and 143 HCC patients after curative treatment (included part of the 113 HCC patients who received curative treatment) were enrolled in the retrospective study. Blood samples were collected for CTC tests from 113 HCC patients before any curative treatment, and data from these patients were used to analyze the correlation of CTC count and clinical characteristics. Forty-five of 113 HCC patients were treated with curative treatment, and data were used to analyze the prognostic value of pre-treated CTC level on HCC recurrence. One hundred and forty-three HCC patients had CTC tests after curative treatment, and data were used to analyze the prognostic value of post-treated CTC level on HCC recurrence. Patients were diagnosed with HCC by pathological examination or non-invasive approach according to World Health Organization criteria. The clinical variables were collected by searching HIS Electronic Medical Record System. Recurrence was diagnosed by typical imaging findings of HCC on computed tomography scans, magnetic resonance imaging, and digital subtraction angiography and raised serum alpha-fetoprotein (AFP) level, with or without histological confirmation. Time to recurrence (TTR) was calculated by the interval between curative treatment and diagnosis of any type of recurrence.

The study was approved by the Ethics Committee of the Jiangmen Central Hospital (Jiangmen, China) and written informed consent was obtained from all participants. This study was conducted in accordance with the World Medical Association Declaration of Helsinki.

### CTC test

Peripheral blood samples (5 ml) were collected for CTC test using the Canpatrol^TM^ CTC analysis system (SurExam, China). Isolation and classification of CTC were performed as previously described^39^. CTC were classified into three phenotypes by multiple mRNA *in situ* hybridization tech1nology: epithelial CTC (epithelial marker^+^/mesenchymal marker^−^/CD45^−^/DAPI^+^ cells), biophenotypic CTC (epithelial marker^+^/mesenchymal marker^+^/CD45^−^/DAPI^+^ cells), and mesenchymal CTC (epithelial marker^−^/mesenchymal marker^+^/CD45^−^/DAPI^+^ cells).

### Statistical analysis

Statistical analyses were performed using SPSS 13.0 (SPSS Inc., Chicago, IL, USA) and GraphPad Prism 5.0 (GraphPad Software, San Diego, CA, USA). Data were presented as mean ± SEM or median (Range). Comparisons between groups were analyzed using a *Chi-squared test*, *Paired t test*, or *Student t test* where appropriate. The correlations between the TTR and CTC count were analyzed using Kaplan-Meier survival curves and a *log-rank test*. All statistical analyses were two-tailed and a *P* value of <0.05 was considered to be statistically significant.

## References

[1] Llovet JM (2016). Hepatocellular carcinoma. Nat Rev Dis Primers.

[2] Kim JU (2016). Hepatocellular carcinoma: Review of disease and tumor biomarkers. World J Hepatol.

[3] Miller MC, Doyle GV, Terstappen LW (2010). Significance of Circulating Tumor Cells Detected by the CellSearch System in Patients with Metastatic Breast Colorectal and Prostate Cancer. J Oncol.

[4] Sun YF (2013). Circulating stem cell-like epithelial cell adhesion molecule-positive tumor cells indicate poor prognosis of hepatocellular carcinoma after curative resection. Hepatology.

[5] Lianidou ES, Strati A, Markou A (2014). Circulating tumor cells as promising novel biomarkers in solid cancers. Crit Rev Clin Lab Sci.

[6] Kalluri R, Weinberg RA (2009). The basics of epithelial-mesenchymal transition. J Clin Invest.

[7] Thiery JP, Acloque H, Huang RY, Nieto MA (2009). Epithelial-mesenchymal transitions in development and disease. Cell.

[8] Yu M (2013). Circulating breast tumor cells exhibit dynamic changes in epithelial and mesenchymal composition. Science.

[9] Lecharpentier A (2011). Detection of circulating tumour cells with a hybrid (epithelial/mesenchymal) phenotype in patients with metastatic non-small cell lung cancer. Br J Cancer.

[10] Armstrong AJ (2011). Circulating tumor cells from patients with advanced prostate and breast cancer display both epithelial and mesenchymal markers. Mol Cancer Res.

[11] Zhao R (2017). Expression and clinical relevance of epithelial and mesenchymal markers in circulating tumor cells from colorectal cancer. Oncotarget.

[12] Li TT (2015). Evaluation of epithelial-mesenchymal transitioned circulating tumor cells in patients with resectable gastric cancer: Relevance to therapy response. World J Gastroenterol.

[13] Chen J, Cao S-W, Cai Z, Zheng L, Wang Q (2017). Epithelial-mesenchymal transition phenotypes of circulating tumor cells correlate with the clinical stages and cancer metastasis in hepatocellular carcinoma patients. Cancer Biomark.

[14] Liu Y-k (2016). An improved strategy to detect the epithelial-mesenchymal transition process in circulating tumor cells in hepatocellular carcinoma patients. Hepatol Int.

[15] Qi LN (2018). Circulating tumor cells undergoing EMT provide a metric for diagnosis and prognosis of patients with hepatocellular carcinoma. Cancer Res.

[16] Ou H (2018). Circulating Tumor Cell Phenotype Indicates Poor Survival and Recurrence After Surgery for Hepatocellular Carcinoma. Dig Dis Sci.

[17] Schulze K (2013). Presence of EpCAM-positive circulating tumor cells as biomarker for systemic disease strongly correlates to survival in patients with hepatocellular carcinoma. Int J Cancer.

[18] Fan ST (2011). Prediction of posthepatectomy recurrence of hepatocellular carcinoma by circulating cancer stem cells: a prospective study. Ann Surg.

[19] Zhang Y, Li J, Cao L, Xu W, Yin Z (2012). Circulating tumor cells in hepatocellular carcinoma: detection techniques, clinical implications, and future perspectives. Semin Oncol.

[20] Chiappini F (2012). Circulating tumor cells measurements in hepatocellular carcinoma. Int J Hepatol.

[21] Vona G (2000). Isolation by Size of Epithelial Tumor Cells: A New Method for the Immunomorphological and Molecular Characterization of Circulating Tumor Cells. Am J Pathol.

[22] Parkinson DR (2012). Considerations in the development of circulating tumor cell technology for clinical use. J Transl Med.

[23] Lemoine A (1997). Prospective evaluation of circulating hepatocytes by alpha-fetoprotein mRNA in humans during liver surgery. Ann Surg.

[24] Witzigmann H (2002). Prospective evaluation of circulating hepatocytes by α-fetoprotein messenger RNA in patients with hepatocellular carcinoma. Surgery.

[25] Kienle P (2000). Detection of isolated disseminated tumor cells in bone marrow and blood samples of patients with hepatocellular carcinoma. Arch Surg.

[26] Attard G, de Bono JS (2011). Utilizing circulating tumor cells: challenges and pitfalls. Curr Opin Genet Dev.

[27] Allard WJ (2004). Tumor Cells Circulate in the Peripheral Blood of All Major Carcinomas but not in Healthy Subjects or Patients With Nonmalignant Diseases. Clin Cancer Res.

[28] Gabriel MT, Calleja LR, Chalopin A, Ory B, Heymann D (2016). Circulating Tumor Cells: A Review of Non-EpCAM-Based Approaches for Cell Enrichment and Isolation. Clin Chem.

[29] Gorges TM (2012). Circulating tumour cells escape from EpCAM-based detection due to epithelial-to-mesenchymal transition. BMC Cancer.

[30] Peeters DJE (2014). Detection and prognostic significance of circulating tumour cells in patients with metastatic breast cancer according to immunohistochemical subtypes. Brit J Cancer.

[31] Schmelzer E, Wauthier E, Reid LM (2006). The phenotypes of pluripotent human hepatic progenitors. Stem Cells.

[32] de Boer CJ, van Krieken JHJM, Janssen-van Rhijn CM, Litvinov SV (1999). Expression of Ep-CAM in normal, regenerating, metaplastic, and neoplastic liver. J Pathol.

[33] Wang S, Zheng Y, Liu J, Huo F, Zhou J (2018). Analysis of circulating tumor cells in patients with hepatocellular carcinoma recurrence following liver transplantation. J Invest Med.

[34] Alix-Panabieres C, Pantel K (2014). Challenges in circulating tumour cell research. Nat Rev Cancer.

[35] Chui MH (2013). Insights into cancer metastasis from a clinicopathologic perspective: Epithelial-Mesenchymal Transition is not a necessary step. Int J Cancer.

[36] Nieto MA (2011). The Ins and Outs of the Epithelial to Mesenchymal Transition in Health and Disease. Annu Rev Cell Dev Bi.

[37] Fischer KR (2015). Epithelial-to-mesenchymal transition is not required for lung metastasis but contributes to chemoresistance. Nature.

[38] Zheng X (2015). Epithelial-to-mesenchymal transition is dispensable for metastasis but induces chemoresistance in pancreatic cancer. Nature.

[39] Wu S (2015). Classification of circulating tumor cells by epithelial-mesenchymal transition markers. PLoS One.

